# The Quality of Meat Derived from Turkey Females Reared Under Extensive Conditions

**DOI:** 10.3390/foods15020195

**Published:** 2026-01-06

**Authors:** Justyna Batkowska, Mirosław Słowiński, Ewa Januś, Małgorzata Karwowska, Antoni Brodacki

**Affiliations:** 1 Institute of Biological Basis of Animal Production, University of Life Sciences in Lublin, 13 Akademicka St., 20-950 Lublin, Poland; justyna.batkowska@up.edu.pl (J.B.); antoni.brodacki@up.edu.pl (A.B.); 2 Department of Food Technology and Assessment, Institute of Food Science, Warsaw University of Life Sciences, 159c Nowoursynowska St., 02-776 Warsaw, Poland; miroslaw_slowinski@sggw.edu.pl; 3 Department of Cattle Breeding and Genetic Resources Conservation, University of Life Sciences in Lublin, 13 Akademicka St., 20-950 Lublin, Poland; 4 Department of Meat Technology and Food Quality, University of Life Sciences in Lublin, 8 Skromna St., 20-704 Lublin, Poland; malgorzata.karwowska@up.edu.pl

**Keywords:** chemical composition, fatty acid profile, slaughter turkeys, technological traits

## Abstract

The aim of this study is to assess the impact of extensive husbandry on slaughter characteristics and turkey meat quality in two utility types. The experiment was divided into two stages: for the first 6 weeks, 200 medium-heavy (MH) and heavy (H) turkey females were kept in intensive rearing conditions and then divided into a control (MHC/HC) and an extensive group (MHE/HE), with five replications in each group (10 birds per replication) for 10 weeks. In E groups, the balanced mixtures were gradually replaced with wheat at 30, 50, and 70% in subsequent feeding periods. Additionally, birds received green fodder (nettles, clover, and alfalfa) and steamed potatoes. After 16 weeks of rearing, birds were slaughtered, their carcasses were dissected, and the meat was assessed for technological traits (pH, L*a*b*, WHC, and tenderness), chemical composition (protein, ash, and fat), fatty acid profile, and sensory analysis. A higher proportion of liver and gizzard and a lower proportion of abdominal fat were found in the E groups, which resulted from more intensive mobility. Meat from these birds was less tender than that from females in the C groups, but it also contained considerably more protein and less fat. Lower values of fatty acid indices such as PI and AI, as well as a higher content of MUFA and a narrower n-6:n-3 ratio in meat from MH birds, indicate a stronger response of these birds to the extensive rearing system and confirm the health-promoting properties of their meat. The sensory evaluation of the meat meets the expectations of modern consumers regarding both the origin and taste of poultry meat.

## 1. Introduction

In recent years, despite significant growth in conventional poultry production, consumers have shown a preference for purchasing poultry meat from more extensive farming systems. This is due to the belief that animal raw materials obtained in this way are of higher quality and have beneficial properties, as well as the fact that they come from animals kept under natural conditions in accordance with animal welfare principles [1]. On the other hand, extensive poultry farming is most often associated with a multi-species flock of colourful birds freely exploiting the grounds of a small farm [2]. However, it should be noted that such a scale of ‘production’ may be sufficient only to meet the needs of a single household. Meanwhile, poultry meat consumption has been on an upward trend for many decades [3], and this also applies to raw materials from alternative poultry farming systems. Therefore, even these systems required a change in the production scale. The size of the flock, regardless of the rearing system, is linked to the production efficiency of the animals themselves, which has an impact on the economics of rearing.

Additionally, attention should be paid to the animal material available on the market, although the use of local breeds in the context of high market demand and obtaining at least satisfactory economic results is very limited. The price for the end consumer is also incomparably high [4]. Hence, producers’ attention is mainly focused on birds, hybrids that are the result of long-term breeding work aimed at increasing body weight and the proportion of valuable elements in the carcass, improving growth rate and feed conversion, and reducing carcass fatness [5] but also increasing egg production [6]. However, it should be remembered that improvement through the selection of certain traits has contributed to the deterioration of others, including animal welfare and resistance to suboptimal environmental or nutritional conditions [7]. This raises doubts whether commercial, fast-growing hybrids will be able to manage in extensive farming conditions [8,9].

Turkeys are the second most popular slaughter birds after chickens, but due to their specific environmental and nutritional requirements, especially during the early stages of rearing, they are generally associated with intensive farming systems [10,11,12]. Farmers are afraid to buy 1-day-old poults, as they are difficult to rear in the first critical period (6 weeks of life) until they are wattled. Fast-growing heavy or medium-heavy turkeys are typically used for commercial meat production, while slow-growing birds are rare and usually intended for small-scale production [13]. However, due to the limited availability of slow-growing birds, typical fast-growing birds are used, most commonly purchased at 6 weeks of age. However, as turkeys are most often sold in cuts rather than whole carcasses, they may meet consumer needs in this regard [14].

When analysing raw materials obtained from alternative poultry farming systems, in addition to their quantity and quality, consumer acceptance should also be taken into account [5]. The intensive farming system is designed towards maximising production with minimal expenditure, resulting in quite universal raw materials. However, due to overcrowding, unsatisfactory zoohygienic conditions, and the lack of natural environmental elements, it is highly controversial. Intensive production must be carefully managed to avoid adverse effects on meat quality or birds’ health, as stress and disease can negate any increase in productivity [13,15]. At the same time, the consumers’ requirements regarding the origin of meat often confront unacceptable sensory experiences caused by the intensity of taste and smell due to the feeding of plant-based feed, especially green fodders. The increased mobility of the birds and their more intensive muscle activity are also important factors, as they result in less tenderness of the meat [16].

The aim of the study was to assess the impact of an extensive rearing and feeding system on slaughter characteristics and on the quality and chemical composition of breast and thigh muscles of turkey hens representing two different utility types (medium-heavy and heavy). The recent literature provides no empirical studies evaluating the combined effects of utility type and rearing system on slaughter traits and meat quality in turkeys, which highlights a clear research gap addressed in the present study.

## 2. Materials and Methods

As the birds were kept as a commercial flock slaughtered at the end of the typical production cycle, no research procedures compromising the welfare of the birds were performed during the rearing, according to the legislation in force (Act of 15 January 2015 on the protection of animals used for scientific or educational purposes; Directive 2010/63/EU of the European Parliament and of the Council of 22 September 2010 on the protection of animals used for scientific purposes), and ethics committee approval for the study was not required. All procedures were carried out in accordance with national legislation and ARRIVE guidelines.

The research material consisted of 200 turkey hybrids belonging to two utility types (exact trade names not provided): medium-heavy (MH) and heavy (H). Birds for the trial were randomly chosen from the entire pool of purchased day-old poults (approx. 4000/batch). The initial body weight of the birds was 58.91 ± 3.80 g and 62.18 ± 4.41 g for medium-weight and heavy birds (*p* ≤ 0.001), respectively. Birds were individually marked with wing tags.

The experiment had two stages: in the 6th week of age, birds were kept in an intensive system of rearing, in accordance with the poult supplier’s recommendations (Aviagen^®^; Midlothian, Scotland). Then birds of each type were randomly divided into a control (C) and an extensive group (E) with five replication groups each (10 birds per replication) for 10 weeks.

The C groups were fed ad libitum with a balanced feed mixture adjusted to their age and nutritional requirements. The composition and nutritive value of all feed mixtures are presented in Table 1.

At the 7th week, birds from groups marked as E (extensive) were moved to an open building with the possibility of going out on all rain-free days, from morning until dusk, to a fenced green run (covered with grass, alfalfa, and nettles). The birds were transferred in May (late spring in Poland) and reared until mid-August (late summer). According to data from the Lublin-Radawiec meteorological station (51°13′, 22°24′), the basic climate indicators (T—temperature, H—humidity, R—sum of rain, and P—atmospheric pressure) in the consecutive months of the experiment were as follows:

May T 8.5–22.4 °C, H 60%, R 17.1 mm, P 988.4 hPa;

June T 18.8–24.2 °C, H 75%, R 95.5 mm, P 985.8 hPa;

July T 15.7–27.2 °C, H 71%, R 92.4 mm, P 986.4 hPa;

August T 14.8–26.7 °C, H 72%, R 43.6 mm, P 987.3 hPa [17].

During the first few days, the birds progressively adapted to the new environmental conditions (natural light, runs). The run area was divided using a movable fence so that birds from different replication subgroups did not mix. The outdoor area (runs) was partially shaded to provide shelter from the sun. Birds had continuous access to the shelter building. The stocking density was 0.2 birds/m^2^ in the outdoor area and 3 birds/m^2^ inside the shelter building. Each box (regardless of rearing system) was equipped with two bell drinkers and two manual feeders, ensuring at least 2 cm of feeding space per bird. Turkeys reared intensively throughout the entire rearing period were kept in accordance with Aviagen^®^ recommendations in a windowless, mechanically ventilated building with a stocking density of 4 birds/m^2^ of litter area (wheat straw). A lighting programme of 16 h of light and 8 h of darkness per day was applied to promote proper bone development.

In the E groups, the balanced mixtures were gradually replaced with wheat at 30, 50, and 70% in subsequent feeding periods. In addition, from the 9th week, group E received 0.1 kg/bird of green fodder consisting of nettles, clover, and alfalfa per day, and from the 13th week, also 0.1 kg/bird of steamed potatoes. Birds had ad libitum access to natural green forage in the range area and were not kept under controlled conditions, allowing for individual feed intake measurements. The nutritional value of final mixtures partially mixed with wheat is presented in Table 2, while that of farm fodders is presented in Table 3. The composition of the vitamin and mineral premix, a component of all feed mixtures used in the experiment (per 1000 g), is shown in Table S1.

After 16 weeks of rearing, four turkey females from each replication were randomly chosen. The birds were slaughtered in a commercial poultry abattoir by decapitation (EU Regulation No. 1099/2009 of 24 September 2009 on the protection of animals at the time of slaughtering), plucked, and eviscerated. Then the carcasses were chilled up to 4 °C inside, and their simplified dissection was performed [18]. The carcass yield (CY) and proportions of giblets and abdominal fat pad (AFP) in the live body weight were calculated, as well as the share of the following carcass cuts: breast muscles (BM), legs (thigh and drumstick), wings, and trunk.

During the dissection analysis, samples of breast (20 pcs) and thigh muscles (20 pcs) were collected, and then the quality traits of meat were determined as follows: acidity 15 min. and 24 h after slaughter (pH metre CP-251); water holding capacity [19]; natural drip loss [20]; and thermal loss [21]. After cooking, cuboid cores (1 cm × 1 cm × 2 cm of edge length) were cut from the heat-treated muscles, parallel to the longitudinal orientation of the muscle fibers. Warner–Bratzler shear force was determined using a texture analyzer TA-XT plus (Stable Micro Systems Ltd., Surrey, UK) equipped with a V-shaped blade. Samples were shorn at a crosshead speed of 2 mm/s.

Colour coordinates were measured on freshly cut muscle surfaces using an X-Rite Colour^®^ Premiere 8200 spectrophotometer (X-Rite Inc., Kentwood, MI, USA). The thickness of the samples was at least 10 mm. The instrumental conditions were a 25.4 mm diameter area aperture. The measurement was carried out in the range of 360–740 nm. The illuminant D65 and a 10° standard colorimetric observer were used as a source of light. A white standard was used as a reference source with a specification of L* = 95.87, a* = −0.49, and b* = 2.39. The results were expressed in units of the CIE L*a*b* [22] system, for which the distinctions reflect, respectively, as follows:

L*—colour lightness, generally adopts positive values and can take values from 0 for an extremely black body to 100 for a perfectly white body;

a*—chromaticity in the red–green range; means red if it is positive, green if it is negative;

b*—yellow–blue chromaticity; means yellow if it is positive, blue if it is negative.

The basic chemical composition of meat from the breast and thigh muscles of turkeys was determined as follows: water content (DM—dry matter) by the drying method according to PN-ISO 1442:2000 [23], total protein content by the Kjeldahl method according to PN–75/A-04018 [24], fat content using the Soxhlet method according to PN-ISO 1444:2000 [25] with a Tecator SOXTEC HTZ-2 (Stable Micro Systems Ltd., Surrey, UK) apparatus, and total ash content according to PN-ISO 936:2000 [26].

The fatty acid profile of breast and thigh meat was analysed using gas chromatography according to PN-EN ISO 5508:1996 [27] and PN-EN ISO 5509:2001 [28] using a Varian 450-GC gas chromatograph (Varian GC, Palo Alto, GA, USA) fitted with a flame ionisation detector. Injector and detector temperatures were 250 °C and 300 °C, respectively. After injection, the column temperature was programmed to increase to 200 °C for 10 min and subsequently increased to 240 °C at the rate of 3 °C/min. Then, the column temperature was held at the final temperature for 4 min. Helium was used as a carrier gas (3 mL/min). The following indices were calculated based on the particular fatty acid concentrations or their groups: PI—peroxidation index [29], AI—atherogenic index, and TI—thrombogenic index [30].

Due to reports of significant variation in the sensory parameters of poultry meat depending on the rearing system, preliminary studies of these relationships were undertaken. Meat samples taken from the breast and thigh muscles of birds during dissection were subjected to 24 h of exposure to a 0.9% aqueous salt solution (physiological saline is used to standardise sample hydration), followed by heat treatment for 90 min at 180 °C. The samples were prepared in an oven on a special cooking grid that has no contact with meat juices. Twenty untrained panellists were selected randomly; all were non-smokers and healthy individuals with a normal sense of smell. Four samples (corresponding to the experimental groups), prepared in another room and coded by numbers, were presented to each panellist. The serving order of samples was randomised for each panellist, and the samples were presented in balanced pairs representing meat from intensively and extensively reared birds. Disposable white plates and water for rinsing the mouth were provided. The panellists were asked to evaluate the following sensory attributes on a scale from 0 (the worst) to 5 (the best): flavour intensity and desirability, taste intensity and desirability, and juiciness and tenderness [31].

The obtained data were statistically analysed using the SPSS 24.0 statistical package [32]. The Shapiro–Wilk test was conducted to verify the normality of the data. The groups were compared using ANOVA and post hoc Tukey’s test. A single bird (randomly chosen from a particular box) was used as replication. The two-factorial analysis of the model, including the utility type (UT) of turkeys and rearing system (RS), as well as the interaction between both factors, was conducted.

## 3. Results

Data on rearing efficiency have been published previously [8]. The main aspects of the turkey meat quality are presented in this paper.

Table 4 presents the results of the slaughter analysis of turkeys divided according to the rearing system and the hybrid type, and the significance of the interaction between these factors is indicated. As expected, the body weight of birds before slaughter was higher in heavy than medium-heavy birds. Interestingly, the response of hybrids of both types to the extensive farming system was different: HC birds weighed more than HE ones, but MHE weighed more than MHC turkeys. In the case of this trait, a significant impact of individual experimental factors and interactions between them was observed (*p* ≤ 0.05). The UT × RS interaction was also significant for slaughter performance, but in this case, the difference between the groups was not as remarkable.

The farming system and the utility type of the turkeys had an impact on the proportion of edible giblets, such as liver and giblets. The percentage of these organs in body weight was greater in birds from extensive groups. In the case of liver and gizzard, the significant effect of UT on birds was also noticed.

In carcasses of heavy turkeys kept extensively, the content of the abdominal fat pad was 40% smaller than in HC birds. Additionally, in medium-heavy birds, regardless of the group, the content of AFP was very low and similar in both groups. A highly significant effect of the utility type on the content of abdominal fat in birds’ carcasses was observed. It is substantial that during carcass evisceration, the presence of abdominal fat was found in 90% of heavy birds and only in 50% of medium-heavy females.

The rearing system and the utility type of the turkeys did not affect the percentage share of particular carcass parts, except for breast muscles, where the rearing system did affect their proportions. However, no statistically significant differences were found between the experimental groups.

The technological traits of breast and thigh muscles, depending on the utility types and rearing system of turkey females, are presented in Table 5 and Table 6, respectively. The pH values of the breast muscles of birds in all groups were similar, with the pH measured 24 h after slaughter being lower than that measured 15 min post mortem, indicating that glycolysis in the muscles proceeded normally. A similar trend was also observed in pH measurements in the thigh muscles.

The lightness (L*) of breast muscles was high regardless of the group of birds, while slightly higher colour saturation to yellow (b*) was observed in the meat of turkeys reared extensively, regardless of muscle type and utility type of birds. The breast and thigh muscles of intensively reared turkeys were characterised by greater saturation to red (a*) compared with the birds from the E groups.

The hybrid type did not considerably affect any of the analysed parameters of both muscles. In the breast muscle, a significant effect of the turkey rearing system on the L*, a*, and b* was found. Meat from extensively reared birds was significantly darker regardless of their UT and was also characterised by a slightly poorer WHC capacity (*p* > 0.05) and greater heat loss (*p* > 0.05). A statistically significant UT × RS interaction was observed for breast muscle lightness (L*) and WHC. In the thigh muscle, a significant effect of the rearing system was observed for lightness (L*) and thermal leakage; in both parameters, the observations made previously were confirmed. A substantial effect of the interaction of both experimental factors on the technological characteristics of thigh muscles was observed in the case of the first pH measurement, lightness, and weight loss after heat treatment.

The greatest tenderness, measured by the lowest force required to cut the muscle fibres, was observed in the breast and thigh muscles of HC turkeys, 4.91 and 10.48 N, respectively. Two regularities were observed in the values of this indicator, i.e., the breast muscles were more tender than the thigh muscles in all groups of turkeys, and both the breast and thigh muscles of birds from extensive RS were less tender than the muscles of intensively farmed turkeys. The differences were not statistically significant, probably due to the high individual variability of traits.

Table 7 presents the basic chemical composition of turkey meat depending on the utility type and rearing system. RS significantly affected the fat content in breast muscle (*p* ≤ 0.01), as well as dry matter, fat, and protein content in thigh muscle (*p* ≤ 0.01). The breast muscles of birds from the HE and MHE groups were characterised by a lower fat content and a slightly higher protein level. Similar relationships were observed in the thigh muscles, while the dry matter level was significantly higher in the muscles of intensively reared birds. A significant effect of hybrid type on meat chemical composition was found for fat (in both muscles) and protein (in thigh muscles); however, no interaction between the experimental factors was observed.

The proportion of particular fatty acids in the breast and the thigh muscle depending on the utility types and rearing system of turkey females included in the experiment is shown in Tables S2 and S3. A more generalised profile of fatty acids in the breast and thigh muscles is presented in Table 8 and Table 9, respectively.

The content of single fatty acids in the breast muscle was only under the impact of the rearing system; however, it was statistically confirmed only in the case of very few acids: 2 saturated (C:12:0 and C:18:0) and 1 monounsaturated (C16:1). The utility type was significant (*p* ≤ 0.05) only in the case of docosapentaenoic acid (C22:5). However, it seems that these observations may be considered to be coincidental, also due to the generally low fat content in the breast muscle of birds.

In the case of thigh muscles, variation caused by the rearing system was observed more frequently, but the relationships between groups were not consistent. Birds from extensively reared groups were characterised by lower myristic (C14:0) acid content, which was also lower in medium-heavy birds than in heavy ones. In the case of monounsaturated acids, significantly higher levels of palmitoleic (C16:1) and oleic acid (C18:1) were observed in the HE and MHE groups, whereas heptadecenoic acid (C17:1) levels were lower. In the same groups, a higher average content of unsaturated acids, i.e., linoleic (C18:2), linolenic (C18:3), and arachidonic (C20:4), and lower levels of eicosadienoic (C20:2) and dihomo-γ-linolenic (C20:3) acids were observed. Docosapentaenoic acid (C22:5) was the only one in which a significant impact of the utility type was noticed. However, because the two turkey types showed opposite responses, this appears to be a circumstantial observation.

No effect of the utility type of hybrids on the fatty acid profile was found in either the breast or thigh muscle of turkeys, nor was there any interaction between the experimental factors (Table 8 and Table 9). The muscles of females from extensive groups were characterised by a lower percentage of SFA and a higher percentage of UFA compared to groups kept under intensive farming conditions.

In the case of the thigh muscle, the relationships between the SFA and UFA groups were similar to those in the breast muscle but were not statistically significant. The muscles of HE and MHE birds contained more monounsaturated fatty acids (MUFA) than the meat of turkeys from the HC and MHC groups. The content of polyunsaturated fatty acids (PUFA) depended on muscle type; numerically, PUFA levels were lower in the thigh muscle than in the breast muscle. In the E groups, PUFA content decreased in the breast muscle and increased in the thigh muscle, regardless of utility type (UT). With respect to the n-6:n-3 ratio, different responses were observed between utility types: in heavy birds, the ratio narrowed, whereas in medium-heavy birds it widened under the influence of extensive feeding, regardless of muscle type.

The rearing system (RS) significantly affected fatty acid indices, although the direction and strength of these effects depended on the muscle analysed. In the breast muscle, birds from the heavy group fed with farm fodders (HE) showed significantly higher PI values and lower TI values than intensively reared birds. In contrast, PI values in the thigh muscle were numerically lower in extensively reared birds. A different response to RS was observed in medium-heavy birds. In the breast muscle, PI was considerably lower in extensively fed birds, with similar AI and TI values, whereas in the thigh muscle, all three indices were numerically lower, with statistical significance mainly for AI.

Due to the lack of a statistically confirmed effect of the hybrid type [33] on the chemical composition and fatty acid profile of meat, this factor was omitted when compiling the results of the sensory analysis. Figures S1 and S2 show the average score for particular indicators of sensory analysis of breast and thigh muscle, respectively. No statistically significant differences were found between meat from the extensively reared groups and the conventional ones; however, the difference is quite clear and shows that meat from birds in the HE/MHE groups was rated higher than that from the HC/MHC groups. It was characterised by a more intense flavour (in both muscles) and taste (in the thigh muscle), without compromising on tenderness, which was similar regardless of whether the birds had access to outdoor runs or not. However, due to the relatively small sample size, this analysis should be treated as a preliminary investigation and needs to be repeated.

## 4. Discussion

Our research indicates that different types of commercial turkeys may respond differently—manifested in final body weight—to the extensive rearing and feeding system used. Also, Ince et al. [34] reported a significant impact of the rearing system (intensive, semi-intensive, and extensive) on the body weight of turkeys. The birds were divided into experimental groups after 8 weeks and kept until 16 weeks of age. The highest body weight at the end of rearing was recorded in birds kept extensively. The significant interaction between type of use (heavy birds vs. medium-heavy birds) and method of maintenance in terms of final body weight was also found by Göppel et al. [35]. However, contrary to our results [8], their results suggested that both genotypes could be suitable for extensive, free-range husbandry. Feeding with the green forage (silage or pasturage) allows birds to compensate for potential amino acid/energy deficits resulting from the lack of a commercial balanced feed mixture. Additionally, it was shown that the use of whole wheat in a turkey feeding may improve performance in terms of daily gain and final body weight. Moreover, an economic advantage may be obtained due to a reduced protein intake and lower feed costs [36].

The statistical differences in the carcass yield were not observed; however, its level was satisfactory, especially in the HE group, higher than presented for BIG 6 (heavy) turkey females [37], also reared under semi-intensive conditions, regardless of sex [38]. The proportions of breast muscle in turkey carcasses in our study were much higher than in the study of Damaziak et al. [39] and similar to those presented by fast-growing males rather than females, even though the rearing period was only one week longer. Göppel et al. [35] stated that the differences between various genotypes were mainly limited to live weight and growth rate, but there were no differences in the relative proportions of valuable carcass cuts, which may explain the lack of significant differences in the proportions of carcass components in our studies. It appears that, in general terms, the impact of the rearing system on the dissection characteristics of birds is very limited. Neither our research nor that of other authors has found any variation in the proportions of most elements in carcasses (except the breast muscle) caused by the rearing system [40,41,42].

Significant differences in liver size, observed as larger livers in birds from the extensively fed groups, may be associated with a greater load of alternative feed ingredients compared with turkeys from group E, which received a complete feed mixture formulated for the needs and digestive capacity of highly productive birds. This phenomenon may be explained by the insoluble fibre (IDF) content in the feed, which can increase the metabolic activity of the liver and consequently its size [43]. This is confirmed by observations regarding the percentage of gizzard in body weight, which tended to be higher in groups fed with wheat and green fodder, probably due to the high content of insoluble fibre in the feed ration [44,45,46].

The influence of the housing system and genotype of turkeys, as well as the interaction between these factors, on the content of the abdominal fat pad was demonstrated by Sarica et al. [42]. The carcasses of fast-growing birds kept in a barn system were more fatty. We did not notice the impact of the rearing system on this trait. The modern commercial turkeys are not excessively fat; however, the variation in AFP can explain from 36 to 48% of the variation in fat content of the carcasses [47]. Fat deposition in the turkey carcass may also depend on the sex of the birds; a significantly higher proportion of AFP is found in females than in males. Our research material consisted only of females, and the proportion of AFP in their body weight was significantly lower than in the study of Melnychuk et al. [48]. We found a highly significant impact of the utility type of turkeys on AFP proportions, but it is difficult to find its confirmation in the literature [49,50,51].

The physicochemical properties of meat should be analysed comprehensively, also about the possible occurrence of meat defects. When analysing the pH of turkey muscles included in the study, it is difficult not to consider this characteristic, together with the relatively high lightness of the meat, as indicative of a PSE defect. It seems that the magnitude of this characteristic is at the threshold of the occurrence of this defect. However, Karakaya et al. [52] show that among various species of slaughter poultry, turkey meat has the lowest pH, and the values, which may indicate the possible occurrence of a defect in broiler chickens, in turkeys, are still within the acceptable range. We did not observe any effect of the housing system on the pH of the breast muscle, as was observed in Saric et al. [53]; however, in the thigh muscle, significantly higher values of this characteristic were observed in meat from E rearing. Similarly, in the study by Solaes et al. [54], the rearing system did not cause pH variability in the breast muscle, whereas in the thigh muscle, the acidity relationship was at the threshold of significance (*p*-value = 0.05) and showed a tendency towards a slightly higher level in birds kept under the organic system. The acidity of poultry meat can be linked to other characteristics of the meat, but also to the characteristics of the birds themselves. The pH of the meat may have a strong impact on its colour, with higher pH values resulting in a darker meat colour [55]. It appears that this trend is visible in our results for the breast muscle.

Meat colour remains one of the most important quality attributes for consumers. At the same time, for producers and processors, it constitutes an indicator of potential meat defects (PSE, DFD), as well as of the proper conduct of slaughter-related procedures and a predictor of the freshness of the raw material [56,57,58]. The colour of meat is the result of several parameters (coordinates) and depends on many factors. In the case of poultry, the first is the species (and/or breed) and sex of birds [59,60], followed by the feeding method, due to the possibility of dietary modification of fat quality, including its colour, and also intramuscular fat. The colour of meat from extensively reared birds results from dietary carotenoid pigments, which are deposited not only in the epidermis and fat depots, but also in intramuscular and intracellular muscle lipids [61]. In our studies, RS caused significant darkening of the colour of the breast muscle. Some of the literature confirms the correlation we have observed [62,63], while other studies have not reported RS modifying the colour coordinate value of meat [54,64,65]. It seems that this may also be due to the high individual variability of birds. Interestingly, conventionally farmed meat evaluated in our study was significantly lighter in colour than that presented, for example, in the study of Werner et al. [66], where an additional significant influence of the turkey hybrid type on meat colour was noticed, possibly due to the large variation in their body weight (UT). Sarica et al. [53] found that the muscles of birds using outdoor runs were more intensely saturated with red colour (a*) and probably as a result of better blood supply to more active muscles (increased myoglobin content) [55]. These discrepancies may be influenced by several factors, including the duration of outdoor access, the type of hybrid, and dietary composition, which can affect myoglobin content and meat redness. Therefore, the observed variation in redness in our study can be explained in the context of these interacting factors.

The water-holding capacity of the poultry meat from the birds included in our research showed only slight variation due to the interaction of the experimental factors used (UT × RS). Other authors point to the variability of this trait caused only by the birds’ sex [53]. The observed effects of the interaction and/or rearing system on the technological characteristics of poultry meat may be associated with the greater space available in free-range systems, which could promote increased bird mobility and potentially contribute to lower fat content in meat [62]. In our previous research [67], we did not confirm statistically significant differences between tenderness values in meat from conventionally and extensively reared turkeys; however, numerically, the meat from birds kept with access to outdoor runs was tougher.

Due to the lowest fat content in meat among other types of poultry, turkeys are also characterised by the lowest drip and thermal loss [60]. However, the impact of the rearing system on these indicators may vary [68]. In the case of fast-growing chickens reared extensively, natural leakage may be lower and thermal leakage higher, regardless of the muscle, compared to meat from conventionally reared birds. The slaughter age of the birds should also be taken into account here [69].

Access to outdoor runs also results in less tender meat, measured by the greater force required to cut through muscle fibres, probably due to the larger diameter, surface area, and density of myofibrils in these muscles [70]. We also confirmed that the muscle fibres of meat from extensively reared birds require greater force to cut, both in turkeys [67] and chickens, although in the latter case, genotype (growth rate), and muscle type (breast/thigh muscle) played a significant role [68,69]. It is also possible that the greater motor activity of birds with access to outdoor runs may be associated with higher collagen content and increased cross-linking in the muscles, which could in turn be related to lower meat tenderness [71,72].

The basic chemical composition of poultry meat shows limited nutritional variability, and observed statistical differences may depend, among other factors, on the sensitivity and precision of the analytical methods used. Bogosavljevic-Boskovic et al. [73] demonstrated the highly significant impact of organic farming on protein content in chicken meat, with an increase in this component observed in both thigh and breast muscles. This phenomenon may be explained by the reduction in fat content in the muscles of birds kept in an extensive system (with outdoor access), which may, in turn, increase protein concentration. In addition, access to outdoor runs may contribute to greater muscle development, resulting in higher protein content [64,70]. Our research seems to confirm this correlation: turkey females from the E groups were characterised by meat with reduced fat and increased protein content.

In our research, the fat content in meat (breast muscle and thigh muscle) did not align closely with the size of the abdominal fat pad, particularly in MH birds, despite a previously reported correlation between abdominal fat pad and carcass fat percentage ranging from 0.60 to 0.69 for turkeys. At the same time, differences between rearing systems were evident in this trait, with birds given access to open-air runs showing lower values of this indicator for both utility types. This relationship is confirmed by the work of other authors [42], also in the case of chickens [70,73,74]. The fat content in meat translates into its caloric value, so it can be indirectly concluded that meat from birds raised in conventional conditions could provide more energy [70,75]. The higher moisture and lower fat and energy values observed in meat from the E groups may suggest that increased motor activity could favour myogenesis over lipogenesis [62]. The fat content in the meat, regardless of the rearing system, was similar to that reported in some studies on birds of a similar genotype [59,76].

Poultry meat has been a frequent target of nutritional modification due to the relative simplicity of dietary modification of the quantity and quality of fat. These changes are mainly focused on alterations in the fatty acid profile, which indirectly determines the oxidative stability (“shelf life”) of meat [77]. In previous studies [78], we have shown that despite feeding with a full-ration diet, even the addition of green fodder causes statistically significant changes in the fatty acid profile. Interestingly, as in the study by Cömert et al. [79], we observed an increase in SFA and a decrease in UFA, with the turkeys being fed plant-based feed only as an additive. In these studies, when plant-based feeds partially replaced balanced complete mixtures, the relationships were reversed; we observed an increase in PUFA content and a decrease in SFA in the meat. The most frequently analysed physiologically active components of raw materials of animal origin, including meat, are polyunsaturated fatty acids (PUFAs) of the n-6 and n-3 groups. PUFA n-3 fatty acids seem to be the most important, considering the number of clinical studies that prove these compounds reduce the risk of several chronic diseases, including cardiovascular diseases [77]. In this study, as well as in the previous one [78], where extensive poultry husbandry and feeding systems were the determining factor, we observed a significant increase in the proportion of n-3 fatty acids in the meat. This may confirm the beneficial effect of green fodder on the lipid profile of poultry meat.

The effect of fatty acids on shelf life stems from the tendency of unsaturated fatty acids to oxidise, leading to rancidity over time. Lipid oxidation products may also promote the oxidation of meat pigments [80]. However, it seems that the potentially lower shelf life of meat from group E should not be judged solely on the basis of unsaturated fatty acid content but also by analysing fatty acid indices such as PI (peroxidability index), which indicates the susceptibility of meat to lipid peroxidation. The atherogenicity index (AI) and thrombogenic index (TI) are commonly used to describe the potential impact of dietary fatty acid profiles on the risk of coronary heart disease. Lower values of these indices are generally considered more desirable, as they reflect a lower proportion of fatty acids associated with atherogenesis and thrombosis [81]. In the present study, PI values depended on both the utility type of birds and the muscle analysed. In MH birds, PI values were lower in meat from extensively reared groups, similar to AI and TI in the breast muscle. In contrast, in heavy birds, PI values in the breast muscle were higher under extensive rearing, further highlighting the divergent response of this index depending on utility type. Taken together, these results suggest that, depending on the utility type and muscle analysed, extensive rearing may be associated with potential health-related advantages, but also with differences in oxidative susceptibility that should be interpreted cautiously. One of the important health indicators is the ratio of n6 to n3 fatty acids, because the diet of Western societies is deficient in n-3 fatty acids, and this ratio is very wide, often ranging from 15:1 to even 25:1, whereas the optimal ratio would be 1:1 [82]. n-3 fatty acids help prevent and treat conditions, some of which are considered civilisation diseases, such as hypertension, diabetes, arthritis, inflammatory disorders, and cancer [83]. Monounsaturated fatty acids can help to reduce blood cholesterol levels, thereby helping to prevent coronary heart disease [84]. It therefore appears that these studies confirm the health benefits of meat from turkeys raised in an extensive system.

The lack of statistical confirmation of differences in the sensory evaluation of meat may be due to the fact that they were relatively small or that the evaluators did not constitute a specially trained panel of sensory judges. In addition, the absence of statistically significant differences should be interpreted with caution, as the sensory assessment was conducted as a preliminary study and may have limited power to detect subtle differences. It has been shown that assessors who have previously been trained in this area are able to detect certain differences in the taste and texture of poultry products, while untrained individuals are unable to recognise which products were obtained using extensive methods and which were produced using conventional ones. Also, regular consumption of meat from intensive farming can reduce the sensitivity of the subsequent assessor’s senses to the slightly different taste qualities of meat from extensive farming [85]. Meat obtained from birds kept in an extensive system is usually considered to be tougher and denser but with a richer flavour [86] compared with meat from conventionally reared birds. Our results also indicate a slightly greater flavour intensity, as well as greater desirability (thigh muscle).

The type of hybrid and its growth rate may also have an impact on the sensory properties of meat. It is believed that the meat of slow-growing birds is less tender than that of birds selected for rapid growth, which is explained by their different slaughter age, as well as lower juiciness and tenderness. The meat of conventional broilers becomes tougher with age, while the opposite is observed in slow-growing birds. As birds age and reach sexual maturity, the intensity of the taste and smell of meat, especially darker meat, also increases [5]. Whereas, in our studies, meat tenderness measured by laboratory methods was indeed numerically worse in birds kept with access to outdoor runs, the differences were not statistically confirmed, possibly due to high individual variability. Furthermore, intensive selection of poultry to improve production results has increased the yield, tenderness (delicacy), and juiciness of meat but has significantly reduced its flavour intensity. Apart from genotype, nutrition is considered to be the main factor influencing the improvement of the sensory characteristics of poultry meat, allowing birds to use grass runs or even pastures or feeding them with green fodder [87]. The sensory evaluation did not reveal clear differences in meat tenderness between the groups, and no consistent indications were observed that would link these sensory results to variation in bird mobility or feeding conditions.

## 5. Conclusions

The slaughter yield of turkey females was similar regardless of the utility type (heavy/medium-heavy) as well as the rearing system (conventional/extensive). Birds kept in the extensive system showed a higher proportion of liver and gizzard and a lower proportion of abdominal fat. These differences may be related to the increased opportunity for movement in green-run systems, although the results do not allow firm conclusions about the underlying mechanisms.

The meat of birds from extensive rearing groups tended to be less tender than the meat of females reared intensively, while also showing higher protein content and lower fat levels. Different responses to the rearing system were observed among birds of different utility types. In medium-heavy birds, the extensive system was associated with more favourable fatty acid profiles, reflected in lower values of indices such as peroxidability (PI), atherogenicity (AI), and thrombogenic index (TI), as well as a higher content of polyunsaturated fatty acids (PUFA). These indicators suggest a fatty acid composition considered more desirable from a nutritional perspective.

Sensory evaluation of the meat did not reveal any significant differences related to the rearing system, which aligns with consumer acceptance, while meat from birds reared extensively meets their current preferences regarding animal welfare. These sensory findings should be interpreted with caution, as the evaluation was performed by a relatively small, untrained panel, which may limit the sensitivity of the assessment and the ability to detect subtle differences between groups.

Our empirical data indicate that modern, intensively selected turkey hybrids may be reared under slightly less intensive conditions, in line with consumer preferences, without clear evidence of negative effects on meat quality. These findings suggest that partial extensification of turkey production may support more welfare-friendly and potentially more sustainable production systems without compromising key carcass or meat quality traits. Moreover, this study contributes to a relatively limited body of research focusing on the extensification of turkey farming, rather than the more commonly studied chickens.

## Figures and Tables

**Table 1:** Composition of feed mixtures used in turkey feeding, depending on the age of the birds.

Item (%) ^1^	PS 0–3 Weeks	S 4–6 Weeks	G1 7–9 Weeks	G2 10–12 Weeks	G3 over 13 Weeks
Maize	12.50	12.50	5.00	9.50	10.00
Wheat	36.69	40.09	55.40	48.40	59.00
Soybean meal	39.00	38.00	32.70	32.50	23.53
Fish meal	5.00	2.50		3.00	
Soybean oil	1.00	1.70	2.60	1.75	3.60
Calcium phosphate	2.05	1.80	1.60	1.70	1.05
Limestone	1.75	1.60	1.00	1.42	1.20
Salt	0.15	0.18	0.20	0.23	0.25
NaHCO_3_	0.13	0.13	0.08	0.10	0.10
Organic acids	0.25	0.25		0.30	
Vitamin–mineral premix with amino acids	1.48	1.25	1.42	1.10	1.27
Nutritive value					
Crude protein (%)	27.01	25.34	23.18	22.40	18.1
Metabolic energy (kcal)	2700	2780	2950	2950	3159
Lysine (%)	1.70	1.60	0.95	1.40	1.20
Methionine (%)	0.60	0.58	0.50	0.50	0.60
Met + Cys (%)	1.00	1.00	0.90	0.90	0.80
Ca (%)	1.20	1.10	1.10	1.00	1.30
P (%)	0.80	0.80	0.80	0.80	0.90
Na (%)	0.15	0.15	0.14	0.14	0.20
Vitamin A (IU)	13,320	11,250	12,780	9900	11,430
Vitamin D_3_ (IU)	3946.7	3333.4	3786.7	2933.4	3386.7
Vitamin E (mg)	44.4	37.5	42.6	33.0	38.1

^1^ PS—prestarter; S—starter; G1—grower 1; G2—grower 2; G3—grower 3.

**Table 2:** Composition of final mixtures used in the feeding of turkeys from extensive (E) groups, depending on the age of the birds.

Item (%) ^1^	70% G1 + 30% Wheat	50% G2 + 50% Wheat	30% G3 + 70% Wheat
Maize	3.50	4.30	3.00
Wheat	67.66	72.68	78.26
Soybean meal	22.89	16.50	12.50
Soybean oil	2.00	2.50	2.50
Calcium phosphate	1.12	0.92	0.70
Limestone	0.70	0.85	0.85
Salt	0.14	0.20	0.22
NaHCO_3_	0.07	0.07	0.07
Vitamin–mineral premix with amino acids	0.92	0.98	0.90

^1^ G1—grower 1; G2—grower 2; G3—grower 3.

**Table 3:** Nutritional value of farm feed used in feeding extensive (E) groups of turkeys (g/1000 g).

Item (%)	Potatoes	Green Fodder
Dry matter (g)	229.75	186.35
Metabolic energy (kcal)	441.25	259.60
Crude protein (g)	19.57	42.80
Crude fat (g)	0.965	7.20
Ash (g)	14.40	23.40
Fibre (g)	183.20	40.45

**Table 4:** The results of simplified dissection depending on the utility types and rearing system of turkey females.

Trait	HC		HE		MHC		MHE		Factors’ Impact (*p*-Value)		
	$\bar{x}$	SD	$\bar{x}$	SD	$\bar{x}$	SD	$\bar{x}$	SD	UT	RS	UT × RS
LBW (kg)	9.48 ^d^	0.594	8.67 ^b^	0.585	7.72 ^a^	0.520	8.79 ^c^	0.543	0.010	0.008	0.009
CY (%)	81.39	1.462	83.71	1.624	80.78	3.318	80.68	2.738	0.158	0.111	0.050
Proportion in live body weight (%)											
Liver	1.387 ^a^	0.119	1.623 ^b^	0.227	1.355 ^a^	0.169	1.396 ^a^	0.091	0.001	0.008	0.115
Heart	0.331	0.040	0.341	0.035	0.339	0.037	0.338	0.034	0.760	0.473	0.774
Gizzard	1.316 ^a^	0.204	1.615 ^b^	0.102	1.300 ^a^	0.192	1.554 ^b^	0.110	<0.001	0.009	0.201
AFP	0.917 ^c^	0.250	0.558 ^b^	0.326	0.125 ^a^	0.047	0.127 ^a^	0.081	<0.001	0.088	0.085
Proportion in carcass (%)											
BM	26.44	1.564	27.19	1.637	25.46	1.872	25.69	1.466	0.009	0.906	0.195
Thighs	16.21	0.716	15.75	0.534	15.55	0.827	16.30	1.361	0.378	0.780	0.296
Drumsticks	12.85	0.732	12.75	0.348	12.92	0.838	12.89	1.128	0.332	0.535	0.803
Wings	12.60	0.473	12.20	0.848	12.78	0.557	12.30	0.789	0.785	0.111	0.981
Trunk	31.90 ^a^	1.133	32.11 ^ab^	1.677	33.29 ^b^	0.880	32.82 ^ab^	1.837	0.090	0.286	0.330

UT—utility type; RS—rearing system; HC—conventional group of heavy turkeys; HE—extensive group of heavy turkeys; MHC—conventional group of medium-heavy turkeys; MHE—extensive group of medium-heavy turkeys; SD—standard deviation; LBW—live body weight; CY—carcass yield; AFP—abdominal fat pad; BM—breast muscles; ^a–d^—means differ significantly at *p* ≤ 0.05.

**Table 5:** The technological traits of breast muscles depending on the utility types and rearing system of turkey females.

Trait	HC		HE		MHC		MHE		Factors’ Impact (*p*-Value)		
	$\bar{x}$	SD	$\bar{x}$	SD	$\bar{x}$	SD	$\bar{x}$	SD	UT	RS	UT × RS
pH1	5.67	0.051	5.65	0.473	5.64	0.039	5.64	0.054	0.255	0.082	0.804
pH2	5.64	0.073	5.58	0.024	5.60	0.048	5.56	0.042	0.549	0.503	0.419
L*	52.14 ^b^	0.757	49.23 ^a^	0.789	50.68 ^b^	1.331	48.72 ^a^	2.233	0.136	0.001	0.047
a*	14.20	0.693	15.30	0.221	14.47	1.048	15.40	0.994	0.610	0.012	0.822
b*	1.46	0.277	0.91	0.421	1.16	0.476	0.66	0.484	0.158	0.012	0.897
WHC (cm^2^)	2.95	1.391	3.70	1.125	2.82	1.435	3.87	1.719	0.977	0.176	0.017
Drip loss (%)	1.15	0.369	0.95	0.246	0.91	0.285	1.23	0.517	0.900	0.707	0.133
Thermal loss (%)	20.70	0.751	22.37	1.303	20.20	0.620	22.60	2.022	0.826	0.003	0.546
Tenderness (N)	4.91	2.04	10.01	7.10	5.47	6.90	12.61	7.93	0.600	0.300	0.480

UT—utility type; RS—rearing system; HC—conventional group of heavy turkeys; HE—extensive group of heavy turkeys; MHC—conventional group of medium-heavy turkeys; MHE—extensive group of medium-heavy turkeys; SD—standard deviation; pH1—15 min after slaughter; pH2—24 hrs after slaughter; WHC—water holding capacity; ^a,b^—means differ significantly at *p* ≤ 0.05.

**Table 6:** The technological traits of thigh muscles depending on the utility types and rearing system of turkey females.

Trait	HC		HE		MHC		MHE		Factors’ Impact (*p*-Value)		
	$\bar{x}$	SD	$\bar{x}$	SD	$\bar{x}$	SD	$\bar{x}$	SD	UT	RS	UT × RS
pH1	5.82 ^a^	0.050	5.91 ^b^	0.076	5.86 ^a^	0.041	5.95 ^b^	0.032	0.093	0.344	0.019
pH2	5.79	0.074	5.71	0.112	5.81	0.041	5.85	0.112	0.171	0.150	0.762
L*	45.89 ^b^	0.936	42.76 ^a^	0.905	46.53 ^b^	1.296	42.81 ^a^	1.899	0.564	<0.001	0.021
a*	16.89	0.479	18.15	1.782	16.34	1.388	16.89	0.919	0.123	0.122	0.525
b*	−0.73	0.150	−1.37	1.248	−0.47	0.604	−2.00	0.529	0.585	0.053	0.197
WHC (cm^2^)	4.27	2.114	3.74	1.670	3.18	1.062	3.23	0.778	0.218	0.729	0.668
Drip loss (%)	0.68	0.205	0.42	0.165	0.78	0.471	0.77	0.487	0.182	0.451	0.459
Thermal loss (%)	31.40 ^a^	1.688	35.11 ^b^	1.613	30.03 ^a^	3.840	35.00 ^b^	2.401	0.527	0.200	0.048
Tenderness (N)	10.48	3.58	16.87	2.58	14.74	6.80	15.39	6.54	0.559	0.156	0.266

UT—utility type; RS—rearing system; HC—conventional group of heavy turkeys; HE—extensive group of heavy turkeys; MHC—conventional group of medium-heavy turkeys; MHE—extensive group of medium-heavy turkeys; SD—standard deviation; pH1—15 min after slaughter; pH2—24 hrs after slaughter; WHC—water holding capacity; ^a,b^—means differ significantly at *p* ≤ 0.05.

**Table 7:** The chemical composition of turkeys’ meat depending on the utility types and rearing system of the turkey females.

Trait (%)	HC		HE		MHC		MHE		Factors’ Impact (*p*-Value)		
	$\bar{x}$	SD	$\bar{x}$	SD	$\bar{x}$	SD	$\bar{x}$	SD	UT	RS	UT × RS
Breast muscle											
Dry matter	27.18	0.768	28.00	2.685	27.17	1.355	26.90	0.933	0.121	0.435	0.125
Ash	1.22	0.278	1.26	0.267	1.33	0.243	1.34	0.248	0.122	0.678	0.804
Fat	1.45 ^b^	0.894	1.22 ^ab^	0.543	1.32 ^b^	0.388	0.82 ^a^	0.266	0.039	0.004	0.280
Protein	24.50	0.583	25.52	2.546	24.52	0.992	24.74	1.048	0.247	0.060	0.225
Thigh muscle											
Dry matter	26.05 ^b^	1.356	25.03 ^a^	0.899	25.66 ^ab^	1.428	25.10 ^ab^	1.428	0.708	0.004	0.330
Ash	1.23	0.130	1.20	0.421	1.18	0.452	1.28	0.353	0.304	0.837	0.126
Fat	4.76 ^c^	1.418	2.67 ^a^	0.901	3.64 ^b^	1.250	2.04 ^a^	0.794	<0.001	<0.001	0.320
Protein	20.06 ^a^	1.358	21.15 ^b^	1.203	20.84 ^ab^	1.323	21.78 ^b^	0.958	0.010	<0.001	0.773

UT—utility type; RS—rearing system; HC—conventional group of heavy turkeys; HE—extensive group of heavy turkeys; MHC—conventional group of medium-heavy turkeys; MHE—extensive group of medium-heavy turkeys; SD—standard deviation; ^a–c^—means differ significantly at *p* ≤ 0.05.

**Table 8:** The fatty acid profile in the breast muscle depending on the utility types and rearing system of turkey females.

Item	HC		HE		MHC		MHE		Factors’ Impact (*p*-Value)		
	$\bar{x}$	SD	$\bar{x}$	SD	$\bar{x}$	SD	$\bar{x}$	SD	UT	RS	UT × RS
SFA	36.20	5.74	34.50	4.54	36.85	6.22	34.42	8.50	0.936	0.179	0.972
UFA	63.81	5.51	65.50	4.55	63.16	6.23	65.59	6.02	0.894	0.247	0.857
MUFA	37.27	4.43	41.20	3.45	36.48	4.77	42.85	9.14	0.779	0.002	0.424
PUFA	26.53	8.50	24.30	6.02	26.68	9.14	22.74	5.47	0.837	0.002	0.718
n6	22.19	8.26	18.79	6.37	20.69	8.60	18.61	5.73	0.555	0.003	0.768
n3	4.08	1.37	5.29	1.19	5.50	2.32	3.95	1.48	0.948	0.007	0.300
n6:n3	5.44 ^b^	1.83	3.55 ^a^	1.27	3.76 ^a^	2.24	4.71 ^ab^	2.32	0.503	0.196	0.014
PI	50.82 ^a^	9.09	57.12 ^b^	8.32	60.51 ^b^	17.29	48.40 ^a^	12.47	0.417	0.001	0.944
AI	0.47	0.15	0.45	0.14	0.48	0.12	0.44	0.05	0.840	0.002	0.920
TI	0.84	0.28	0.73	0.19	0.79	0.30	0.79	0.13	0.976	0.006	0.460

UT—utility type; RS—rearing system; HC—conventional group of heavy turkeys; HE—extensive group of heavy turkeys; MHC—conventional group of medium-heavy turkeys; MHE—extensive group of medium-heavy turkeys; SFA—saturated fatty acids; UFA—unsaturated fatty acids; MUFA—monounsaturated fatty acids; PUFA—polyunsaturated fatty acids; PI—peroxidation index; AI—atherogenic index; TI—thrombogenic index; SD—standard deviation; ^a,b^—means differ significantly at *p* ≤ 0.05.

**Table 9:** The fatty acid profile in the thigh muscle depending on the utility types and rearing system of turkey females.

Item	HC		HE		MHC		MHE		Factors’ Impact (*p*-Value)		
	$\bar{x}$	SD	$\bar{x}$	SD	$\bar{x}$	SD	$\bar{x}$	SD	UT	RS	UT × RS
SFA	40.83	5.66	37.36	4.28	41.93	3.17	37.47	6.79	0.736	0.004	0.784
UFA	59.17	5.64	62.65	4.28	58.07	3.18	62.53	6.98	0.734	0.004	0.785
MUFA	36.98	3.67	40.02	2.96	39.86	4.96	41.57	2.48	0.120	0.098	0.634
PUFA	22.19	8.35	22.64	4.20	18.20	6.36	20.96	5.29	0.234	0.498	0.623
n6	17.34	7.73	17.56	4.78	14.51	5.58	17.28	5.08	0.070	0.049	0.648
n3	4.44	1.84	4.84	2.04	3.35	1.50	3.46	1.99	0.870	0.016	0.827
n6:n3	3.91	2.81	3.63	2.71	4.33	2.24	4.99	2.31	0.304	0.482	0.731
PI	56.82	19.57	55.96	18.32	44.86	14.74	43.65	16.81	0.063	0.870	0.978
AI	0.59	0.15	0.51	0.09	0.61	0.09	0.49	0.15	0.865	0.013	0.909
TI	0.98 ^ab^	0.31	0.84 ^a^	0.12	1.10 ^b^	0.21	0.92 ^ab^	0.38	0.296	0.083	0.856

UT—utility type; RS—rearing system; HC—conventional group of heavy turkeys; HE—extensive group of heavy turkeys; MHC—conventional group of medium-heavy turkeys; MHE—extensive group of medium-heavy turkeys; SFA—saturated fatty acids; UFA—unsaturated fatty acids; MUFA—monounsaturated fatty acids; PUFA—polyunsaturated fatty acids; PI—peroxidation index; AI—atherogenic index; TI—thrombogenic index; SD—standard deviation; ^a,b^—means differ significantly at *p* ≤ 0.05.

## Data Availability

The original contributions presented in this study are included in the article/Supplementary Materials. Further inquiries can be directed to the corresponding author.

## Supplementary Materials

The following supporting information can be downloaded at: https://www.mdpi.com/article/10.3390/foods15020195/s1, Table S1: The composition of the vitamin and mineral premix, a component of all feed mixtures used in the experiment (per 1000 g); Table S2: The proportion of fatty acids in the breast muscle depending on the utility types and rearing system of turkey females included in the experiment; Table S3: The proportion of fatty acids in the thigh muscle depending on the utility types and rearing system of turkey females included in the experiment; Figure S1: Average score for particular indicators of sensory analysis of turkey breast meat regardless of utility type; Figure S2: Average score for particular indicators of sensory analysis of turkey thigh meat regardless of utility type.

## Abbreviations

The following abbreviations are used in this manuscript:

MH	medium-heavy
H	heavy
E	extensive (system)
C	conventional (intensive system)
MHC/HC	medium-heavy/heavy control/conventional groups
MHE/HE	medium-heavy/heavy extensive groups
UT	utility type
RS	rearing system
PS	prestarter feed mixture
S	starter feed mixture
G1	grower 1 feed mixture
G2	grower 2 feed mixture
G3	grower 3 feed mixture
DM	dry matter
LBW	live body weight
CY	carcass yield
AFP	abdominal fat pad
BM	breast muscle
WHC	water holding capacity
L*	colour lightness
a*	chromaticity in the red–green range
b*	yellow–blue chromaticity
PI	peroxidation index
AI	atherogenic index
TI	thrombogenic index
SFA	saturated fatty acids
UFA	unsaturated fatty acids
MUFA	monounsaturated fatty acids
