# Supplementary material for: The Quality of Meat Derived from Turkey Females Reared Under Extensive Conditions
Source: Foods. 2026 Jan 6;15(2):195. doi: 10.3390/foods15020195 (PMC12840003; doi:10.3390/foods15020195)
Supplement: Supplementary file 1 [file foods-15-00195-s001.zip › foods-4012648-supplementary.pdf]

foods

MDPI

The quality of meat derived from turkey females reared under extensive conditions

Justyna Batkowska, Mirosław Słowiński, Ewa Januś, Małgorzata Karwowska and Antoni Brodacki

Table S1. The composition of the vitamin and mineral premix, a component of all feed mixtures used in the experiment (per 1000 g).

Item	Content
Crude protein (%)	25
Metabolic energy (kcal)	1250
Sodium (%)	8,8
Calcium (%)	4,5
Phosphorus (%)	3,3
Lysine (%)	12,4
Methionine (%)	15
Met+Cys (%)	15,1
Vitamin A (IU)	900 000
Vitamin D_3 (IU)	266 670
Vitamin E (mg)	3000
Vitamin K_3 (mg)	233
Vitamin B_1 (mg)	133
Vitamin B_2 (mg)	533
Nicotinic acid (mg)	4667
Pantothenic acid (mg)	1000
Vitamin B_6 (mg)	333
Vitamin B_{12} (mcg)	1667
Biotin (mcg)	23 334
Choline chloride (mg)	40 000
Folic acid (mg)	100
Ferrum (mg)	5333
Manganese (mg)	8000
Copper (mg)	2667
Zinc (mg)	6000
Iodine (mg)	100
Cobalt (mg)	27
Selenium (mg)	20

Table S2. The proportion of fatty acids in the breast muscle depending on the utility types and rearing system of turkey females included in the experiment.

Trait	HC		HE		MHC		MHE		Factors' impact (*p-value*)		
	$\bar{x}$	*SD*	$\bar{x}$	SD	$\bar{x}$	SD	$\bar{x}$	SD	UT	RS	UT × RS
Saturated fatty acids											
C12:0	0.121[b]	*0.036*	0.069[a]	*0.031*	0.142[b]	*0.075*	0.054[a]	*0.023*	0.853	<0.001	0.318
C14:0	0.540	*0.403*	0.543	*0.353*	0.639	*0.391*	0.356	*0.185*	0.726	0.270	0.260
C15:0	0.173	*0.067*	0.199	*0.046*	0.271	*0.292*	0.144	*0.053*	0.719	0.403	0.214
C16:0	27.289	*4.912*	26.987	*4.220*	27.566	*4.862*	27.227	*2.065*	0.911	0.625	0.798
C17:0	0.237	*0.137*	0.210	*0.059*	0.294	*0.322*	0.167	*0.027*	0.917	0.274	0.473
C18:0	7.656[b]	*0.789*	6.353[a]	*0.613*	7.763[b]	*1.969*	6.323[a]	*1.100*	0.933	0.006	0.882
C20:0	0.180	*0.062*	0.140	*0.057*	0.172	*0.120*	0.146	*0.043*	0.968	0.255	0.812
Monounsaturated fatty acids											
C16:1	5.828[a]	*0.749*	9.866[b]	*1.306*	6.215[a]	*2.601*	10.356[b]	*1.404*	0.480	<0.001	0.931
C17:1	1.038[a]	*1.653*	0.100[c]	*0.057*	0.317[b]	*0.382*	0.109[c]	*0.031*	0.268	0.079	0.257
C18:1	30.156	*2.873*	30.776	*2.828*	29.687	*3.013*	31.974	*2.792*	0.723	0.164	0.420
C20:1	0.251	*0.104*	0.461	*0.520*	0.259	*0.073*	0.407	*0.453*	0.839	0.124	0.786
Polyunsaturated fatty acids											
C18:2	19.438	*9.154*	15.891	*6.281*	17.480	*8.521*	15.867	*5.226*	0.718	0.350	0.725
C18:3	1.751	*0.898*	1.398	*0.604*	1.603	*1.119*	1.195	*0.527*	0.568	0.221	0.928
C18:4	0.270	*0.230*	0.223	*0.302*	0.493	*0.430*	0.186	*0.184*	0.406	0.119	0.247
C20:2	0.251	*0.119*	0.170	*0.082*	0.213	*0.205*	0.170	*0.048*	0.696	0.208	0.698
C20:3	0.193	*0.041*	0.206	*0.058*	0.201	*0.231*	0.109	*0.081*	0.367	0.423	0.288
C20:4	0.881	*0.463*	0.912	*0.357*	0.637	*0.511*	0.703	*0.539*	0.187	0.775	0.915
C22:4	1.423	*0.624*	1.607	*1.090*	2.157	*1.128*	1.757	*0.906*	0.201	0.752	0.394
C22:5	1.299[a]	*0.459*	2.631[ab]	*0.875*	3.001[b]	*2.281*	1.963[ab]	*1.096*	0.031	0.774	0.270
C22:6	1.028	*0.668*	1.259	*0.564*	0.892	*1.308*	0.790	*0.980*	0.379	0.850	0.626

C12:0 – lauric acid, C14:0 – myristic acid, C15:0 – pentadecylic acid, C16:0 – palmitic acid, C17:0 – margaric acid, C18:0 – stearic acid, C20:0 – arachidic acid, C16:1 – palmitoleic acid, C17:1 – heptadecenoic acid, C18:1 – oleic acid, C20:1 – gondoic acid, C18:2 – linoleic acid, C18:3 – linolenic acid, C20:2 – eicosadienoic acid, C20:3 – dihomo-γ-linolenic acid, C20:4 – arachidonic acid, C22:4 – docosatetraenoic acid, C22:5 – docosapentaenoic acid, C22:6 – docosahexaenoic acid; UT – utility type, RS – rearing system, HC – conventional group of heavy turkeys, HE – extensive group of heavy turkeys, MHC – conventional group of medium-heavy turkeys, MHE – extensive group of medium-heavy turkeys, SD – standard deviation; [a - c] – means differ significantly at $p \leq 0.05$

Table S3. The proportion of fatty acids in the thigh muscle depending on the utility types and rearing system of turkey females included in the experiment.

**Trait**	**HC**		**HE**		**MHC**		**MHE**		**Factors' impact (*p-value*)**		
	$\bar{x}$	*SD*	$\bar{x}$	SD	$\bar{x}$	SD	$\bar{x}$	SD	UT	RS	UT × RS
Saturated fatty acids											
C12:0	0.151	*0.076*	0.087	*0.051*	0.224	*0.306*	0.077	*0.071*	0.641	0.120	0.533
C14:0	0.847[b]	*0.411*	0.501[a]	*0.349*	0.790[b]	*0.338*	0.398[a]	*0.263*	0.034	0.017	0.856
C15:0	0.210	*0.079*	0.229	*0.068*	0.260	*0.205*	0.203	*0.058*	0.791	0.686	0.428
C16:0	31.31	*3.552*	29.65	*3.413*	31.90	*2.565*	29.05	*4.209*	0.994	0.074	0.628
C17:0	0.264	*0.127*	0.201	*0.082*	0.230	*0.058*	0.192	*0.096*	0.519	0.146	0.718
C18:0	7.878	*1.976*	6.549	*1.681*	8.320	*0.973*	7.373	*2.529*	0.332	0.087	0.768
C20:0	0.164	*0.062*	0.134	*0.108*	0.206	*0.122*	0.178	*0.038*	0.212	0.396	0.971
Monounsaturated fatty acids											
C16:1	6.561[a]	*1.262*	9.809[b]	*1.873*	8.294[ab]	*2.937*	10.070[b]	*2.954*	0.250	0.001	0.394
C17:1	0.913[b]	*1.146*	0.551[ab]	*0.593*	0.620[ab]	*0.526*	0.098[a]	*0.028*	0.170	0.017	0.765
C18:1	29.291	*4.082*	29.382	*1.790*	30.641	*3.032*	31.150	*1.784*	0.160	0.037	0.848
C20:1	0.214[a]	*0.084*	0.273[ab]	*0.081*	0.309[b]	*0.065*	0.250[ab]	*0.068*	0.193	0.991	0.083
Polyunsaturated fatty acids											
C18:2	12.741	*7.819*	14.239	*4.784*	11.212	*5.505*	15.098	*4.373*	0.877	0.002	0.583
C18:3	0.887	*0.664*	0.896	*0.626*	0.623	*0.536*	1.023	*0.461*	0.747	0.030	0.355
C18:4	0.419	*0.245*	0.234	*0.192*	0.347	*0.185*	0.218	*0.223*	0.572	0.051	0.715
C20:2	0.360[b]	*0.332*	0.221[ab]	*0.157*	0.397[b]	*0.175*	0.115[a]	*0.043*	0.663	0.012	0.364
C20:3	0.218[b]	*0.100*	0.123[a]	*0.049*	0.162[ab]	*0.116*	0.108[a]	*0.032*	0.284	0.465	0.524
C20:4	0.430	*0.519*	0.523	*0.378*	0.386	*0.385*	0.515	*0.314*	0.863	0.028	0.904
C22:4	3.589	*3.475*	2.459	*2.845*	2.352	*1.668*	1.447	*0.586*	0.219	0.264	0.901
C22:5	2.778	*2.076*	2.964	*1.920*	1.729	*0.824*	1.542	*0.626*	0.032	0.999	0.736
C22:6	0.771	*0.789*	0.977	*0.979*	0.996	*0.964*	0.895	*1.703*	0.858	0.896	0.700

C12:0 – lauric acid, C14:0 – myristic acid, C15:0 – pentadecylic acid, C16:0 – palmitic acid, C17:0 – margaric acid, C18:0 – stearic acid, C20:0 – arachidic acid, C16:1 – palmitoleic acid, C17:1 – heptadecenoic acid, C18:1 – oleic acid, C20:1 – gondoic acid, C18:2 – linoleic acid, C18:3 – linolenic acid, C20:2 – eicosadienoic acid, C20:3 – dihomo-γ-linolenic acid, C20:4 – arachidonic acid, C22:4 – docosatetraenoic acid, C22:5 – docosapentaenoic acid, C22:6 – docosahexaenoic acid; UT – utility type, RS – rearing system, HC – conventional group of heavy turkeys, HE – extensive group of heavy turkeys, MHC – conventional group of medium-heavy turkeys, MHE – extensive group of medium-heavy turkeys, SD – standard deviation; [a - b] – means differ significantly at $p \leq 0.05$

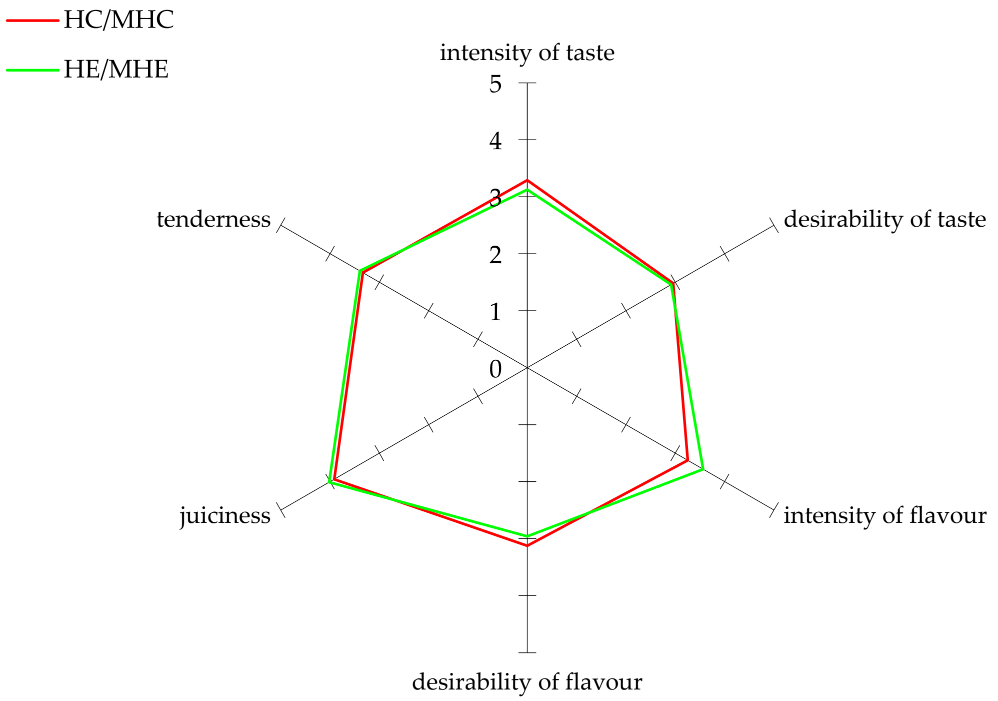

Figure S1. Average score for particular indicators of sensory analysis of turkey breast meat regardless of utility type. HC – conventional group of heavy turkeys, HE – extensive group of heavy turkeys, MHC – conventional group of medium-heavy turkeys, MHE – extensive group of medium-heavy turkeys

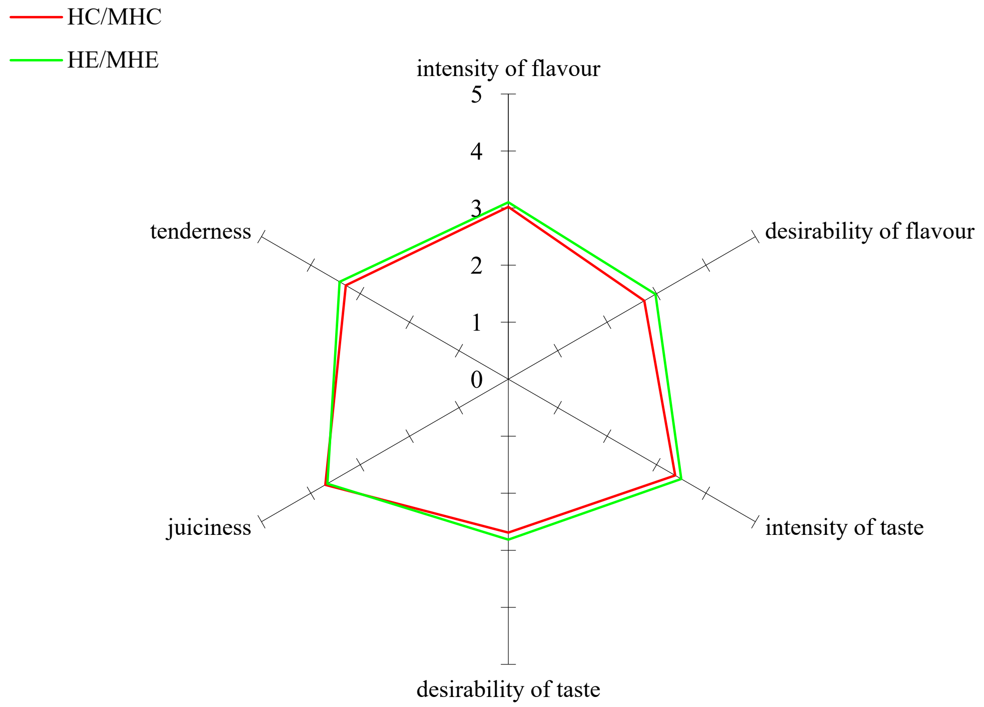

Figure S2. Average score for particular indicators of sensory analysis of turkey thigh meat regardless of utility type. HC – conventional group of heavy turkeys, HE – extensive group of heavy turkeys, MHC – conventional group of medium-heavy turkeys, MHE – extensive group of medium-heavy turkeys
